# Dynamic omnidirectional adhesive microneedle system for oral macromolecular drug delivery

**DOI:** 10.1126/sciadv.abk1792

**Published:** 2022-01-05

**Authors:** Wei Chen, Jacob Wainer, Si Won Ryoo, Xiaoyue Qi, Rong Chang, Jason Li, Seung Ho Lee, Seokkee Min, Adam Wentworth, Joy E. Collins, Siddartha Tamang, Keiko Ishida, Alison Hayward, Robert Langer, Giovanni Traverso

**Affiliations:** 1 Department of Mechanical Engineering, Massachusetts Institute of Technology, Cambridge, MA 02139, USA.; 2 David H. Koch Institute for Integrative Cancer Research, Massachusetts Institute of Technology, Cambridge, MA 02139, USA.; 3 Division of Gastroenterology, Department of Medicine, Brigham and Women’s Hospital, Harvard Medical School, Boston, MA 02115, USA.; 4 Division of Comparative Medicine, Massachusetts Institute of Technology, Cambridge, MA 02139, USA.; 5 Department of Chemical Engineering, Massachusetts Institute of Technology, Cambridge, MA 02139, USA.

## Abstract

Oral drug administration remains the preferred route for patients and health care providers. Delivery of macromolecules through this route remains challenging because of limitations imposed by the transport across the gastrointestinal epithelium and the dynamic and degradative environment. Here, we present the development of a delivery system that combines physical (microneedle) and nonphysical (enhancer) modes of drug delivery enhancement for a macromolecule in a large animal model. Inspired by the thorny-headed intestinal worm, we report a dynamic omnidirectional mucoadhesive microneedle system capable of prolonged gastric mucosa fixation. Moreover, we incorporate sodium *N*-[8-(2-hydroxybenzoyl) amino] caprylate along with semaglutide and demonstrate enhanced absorption in swine resistant to physical displacement in the gastric cavity. Meanwhile, we developed a targeted capsule system capable of deploying intact microneedle-containing systems. These systems stand to enable the delivery of a range of drugs through the generation and maintenance of a privileged region in the gastrointestinal tract.

## Teaser

We developed a dynamic omnidirectional adhesive microneedle system that improves the oral delivery of macromolecules.

## INTRODUCTION

Recombinant human peptides and proteins, such as insulin analogs and glucagon-like peptide-1 (GLP-1) receptor agonists, have expanded the options in treatment for patients with diabetes mellitus (*1*–*3*). Glycemic management of outpatients is predominantly influenced by patients’ level of drug adherence. It has been estimated that more than 50% of treatment failure among patients with diabetes is a result of poor patient adherence with prescribed treatment regimens, which typically involve frequent and repetitive administration of drugs (*4*, *5*). Oral delivery of peptides and proteins is particularly desirable because it avoids the need for injections. Oral delivery of biologics stands to transform how patients are able to receive their therapy. Moreover, oral delivery can transform the quality of life of patients through the mitigation of injectable therapeutics (*6*–*8*). As a result, oral delivery serves as a convenient dosage form for the patient and health care provider and can result in optimal therapeutic outcomes (*9*–*12*). However, the efficacy of orally delivered peptides and proteins is limited because of the susceptibility of the compounds to proteolytic degradation in the gastrointestinal (GI) tract and the poor penetration of the relatively large molecules across the epithelium (*13*, *14*). Several approaches, such as adding protease inhibitors or penetration enhancers to drug formulations and nano/microtechnology solutions, are under preclinical and clinical evaluation (*15*, *16*), which may open new opportunities in the field of oral drug delivery.

Recently, the first oral GLP-1 receptor agonist, which coformulated semaglutide with the penetration enhancer sodium *N*-[8(2-hydroxybenzoyl) amino] caprylate (SNAC) in a tablet form, was approved for the treatment of type 2 diabetes (*17*–*19*). Notably, SNAC has been shown to exhibit buffering action to increase local pH values in the stomach, resulting in lower protease activity and higher peptide stability. Moreover, SNAC is able to promote the monomerization of semaglutide to improve the drug’s solubility and absorption via the transcellular route (*17*, *18*). Nevertheless, quick erosion of the pill (average disintegration time is around 57 min) and its inability to attach to the tissue may result in a short residence time of the tablet and contribute to the low bioavailability of the drug (approximately 1.22 ± 0.25%). Therefore, a relatively large dose (10 mg or more) and high dosing frequency (once daily) are required for oral dosage forms compared to a once-weekly subcutaneous injection of semaglutide (1 mg per week); this may reduce patient adherence and increase treatment cost (*17*, *18*). Contact time and distance from oral formulations or devices have been previously correlated with improvements in drug efficacy and reduce the need for high-frequency administration (*20*–*22*), thus motivating the development of systems capable of supporting transient immobilization. Currently, the most commonly used materials for this purpose are either too quick or too slow to adhere to tissue; they may also release toxic chemicals to induce inflammation (*23*). The development of strong GI adhesive systems that have low chemical reactivity, exhibit an appropriate adhesive speed and strength, can naturally degrade with minimal tissue damage, have a low risk of causing infection or inflammation, and do not occupy too much space in the GI cavity represents a significant unmet need in the field (*24*, *25*).

Chemical-based adhesives, such as acrylic acid derivatives, cellulose derivatives, and alginate, need an abundant amount of hydrophilic functional groups (e.g., carboxyl and hydroxyl groups) to entangle and penetrate mucin: Hydrophilic functional groups enable the formation of numerous hydrogen bonds between the polymer and mucin (*26*). However, excess hydration may result in slippery mucilage formation, which could lower mucoadhesion (*26*). The presence of specific surface functional groups (e.g., COOH, NH_2_, or SH) is necessary for significant adhesion; however, covalent interactions can significantly alter surface biomolecules, which increases the risk of inflammation (*27*). In nature, more than 1500 species of thorny-headed worms act as permanent parasites in the intestine of vertebrates (e.g., fish) (*28*). These worms attach in the gut of their host with the help of cylindrical, globular, and retractable thorny proboscis. At the attachment site, the proboscis penetrates the epithelium of adjacent villi using hooks, which firmly anchor the worms in place (*29*). Inspired by this smart adhesive strategy, micrometer-sized hooks may be used to practically and controllably induce safe and durable mucoadhesion. Such a strategy could enable a device to firmly anchor itself to the GI wall without causing significant damage, supporting the potential to transform formulation-tissue interaction and thus enhance absorption.

**Fig. 1.**
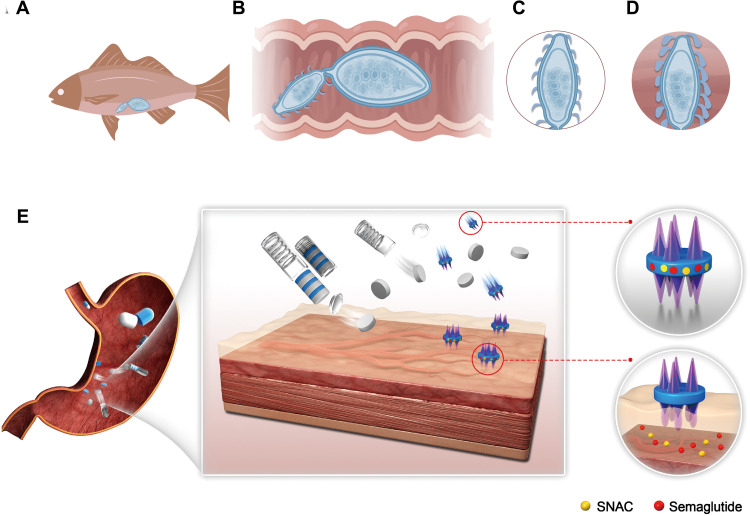
Scheme of DOAMS design and actuation. (**A** and **B**) Thorny-headed worm adheres to the intestine of the fish. (**C** and **D**) The microneedle-like structure on the head of the worm swells after penetrating the intestine to provide strong adhesion. (**E**) After ingestion, the jack-in-the-box device would spontaneously respond to the low-pH environment in the stomach and deploy the engineered tablets to the tissue. The DOAMS would then penetrate the tissue and securely anchor itself in the mucus by the self-triggered extension. In this way, the drug could be gradually released specifically to the tissue surface for a relatively long time.

Another challenge for oral delivery is that the formulation can be prematurely exposed to the harsh GI environment, which can cause it to break down before it reaches its intended target (*30*). A gelatin capsule is commonly used to deliver oral formulations to avoid premature exposure of the formulations (*31*). Unfortunately, when gelatin dissolves, it becomes quite adherent, which can foul and interfere with the release of the formulation to the tissue surface. A self-triggered device, which can help transport the delivery system and support an optimal interaction between the formulation and the GI mucus, is one potential solution. Here, we describe a design inspired by the “jack-in-the-box” system, which acts as a smart capsule to deliver a tablet to the stomach, and then upon triggering by the local gastric environment, it ejects the microneedle-containing tablets into the gastric cavity, inducing appropriate contact with a specialized tablet. The tablet has been engineered with cylinder-shaped cavities on both sides, which house the dynamic omnidirectional adhesive microneedle system (DOAMS). After the tablet is ejected and subsequently comes into contact with the tissue, the DOAMS, mimicking the natural thorny-headed worm, endows the classic tablets with strong adhesive properties to adhere to the tissue surface. The DOAMS has transient mucosal fixation properties while inducing minimal damage to the tissue (Fig. 1). We believe that this bioinspired strategy could be used to add a micrometer-sized modification to traditional dosage forms while remarkably improving their therapeutic performance.

## RESULTS

### The core-shell structure of the DOAMS microneedles

**Fig. 2.**
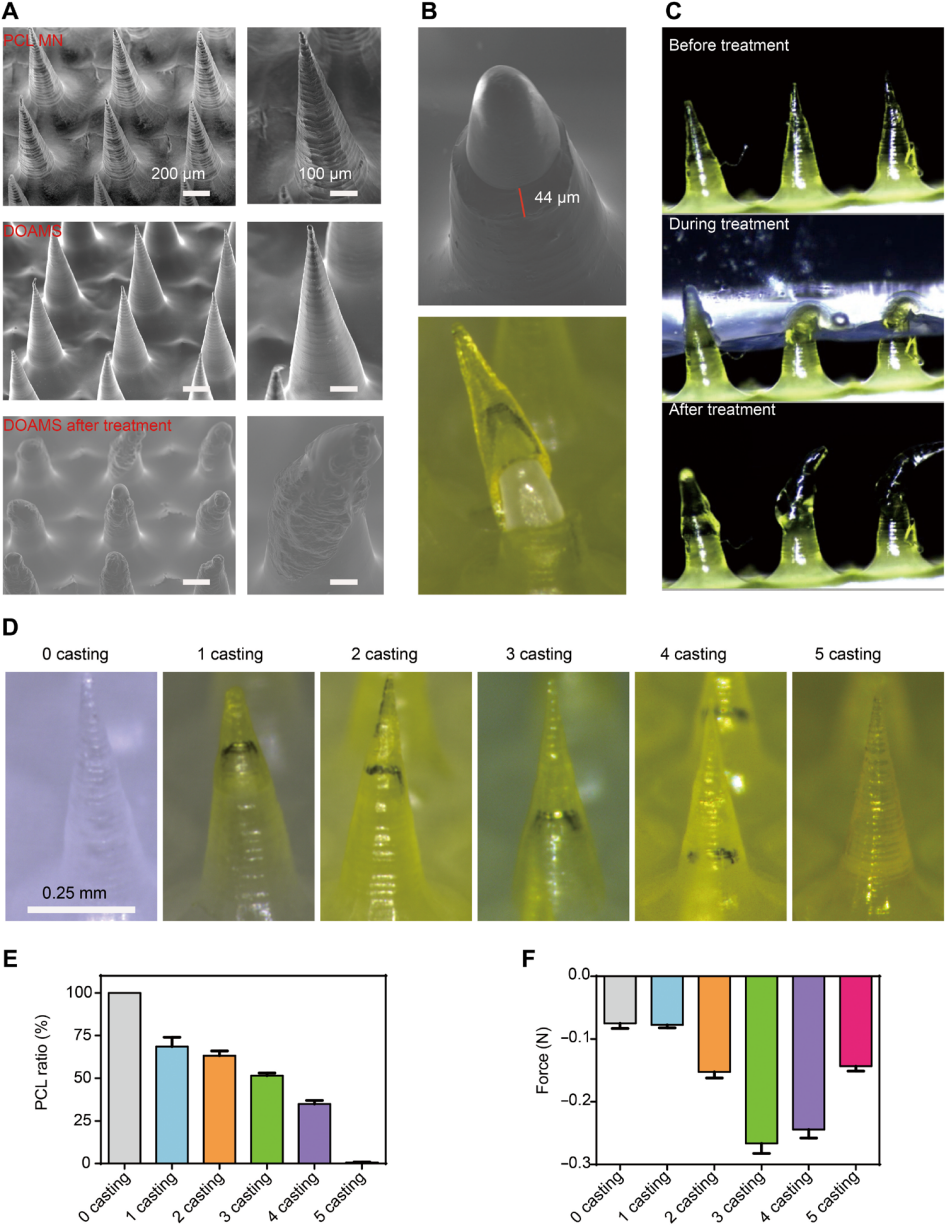
Characterization of DOAMS. (**A**) SEM images of PCL and DOAMS MNs (before and after swelling). Photo credit: Wei Chen, MIT. (**B**) A single MN showing the double-layered structure and the thickness of the outer layers. Photo credit: Wei Chen, MIT. (**C**) Microscope images of DOAMS MNs expanding in a hydrogel. Photo credit: Wei Chen, MIT. (**D**) Images showing the boundary between the inner and outer layers for the different number of Carbopol casting layers. Photo credit: Wei Chen, MIT. (**E**) As the number of Carbopol casting layers increased, the ratio of PCL decreased. (**F**) The adhesive force of the DOAMS for each of the different number of Carbopol casting layers. Data represent means ± SD (*n* = 5).

To build smart microneedles capable of both extending and penetrating tissue, we designed biphasic core-shell structures with a soft outer layer and a rigid inner core. Carbopol, a commercial mucoadhesive polymer, which is strongly mucoadhesive when wet, was selected as the outer layer; polycaprolactone (PCL; molecular weight, 45 kDa), a thermoplastic polyester, was used for the inner core. The DOAMS had a yellowish tint due to the addition of 5% fluorescein isothiocyanate (FITC) in the Carbopol solution (fig. S1); the uncasted PCL microneedles (PCL MNs) were not tinted (white). When viewed with a scanning electron microscope (SEM), the surface of the DOAMS appeared to be relatively less textured and smoother compared with the PCL MN (Fig. 2A). This is likely due to the higher charging effect (electrons are trapped, and a local electrical potential is formed) of Carbopol compared with PCL. When the DOAMS microneedles were cut at their base, a pliable shell with a thickness of around 44 μm and an inflexible conical PCL core was observed (Fig. 2B). Notably, the DOAMS microneedles were effortlessly able to penetrate homogeneous agarose hydrogel (they could also perforate stomach mucous; fig. S2). After penetrating the gel, the DOAMS microneedles autonomously changed their shape (Fig. 2C and movie S1), which suggests that they underwent spontaneous swelling and extension. The capability of the microneedles to penetrate and extend mainly depended on the volumetric ratio of the outer and inner materials. This ratio depended on the number of Carbopol coating layers on the PCL MNs, which varied from 1 to 5: As the number of casting rounds increased, more Carbopol occupied the space in the needle cavity of the polydimethylsiloxane (PDMS) mold (Fig. 2D), which resulted in diminishing amounts of PCL (Fig. 2E). Structures with the highest adhesion strength balanced penetration and extension capabilities: It was found that three castings were the optimal condition to fabricate the strongest adhesive candidate (Fig. 2F). This investigation demonstrated that a biomimetic core-shell microneedle design with adjustable adhesive capability could be used to improve material-tissue interactions.

### Dynamic adhesion of the DOAMS on swine stomach

**Fig. 3.**
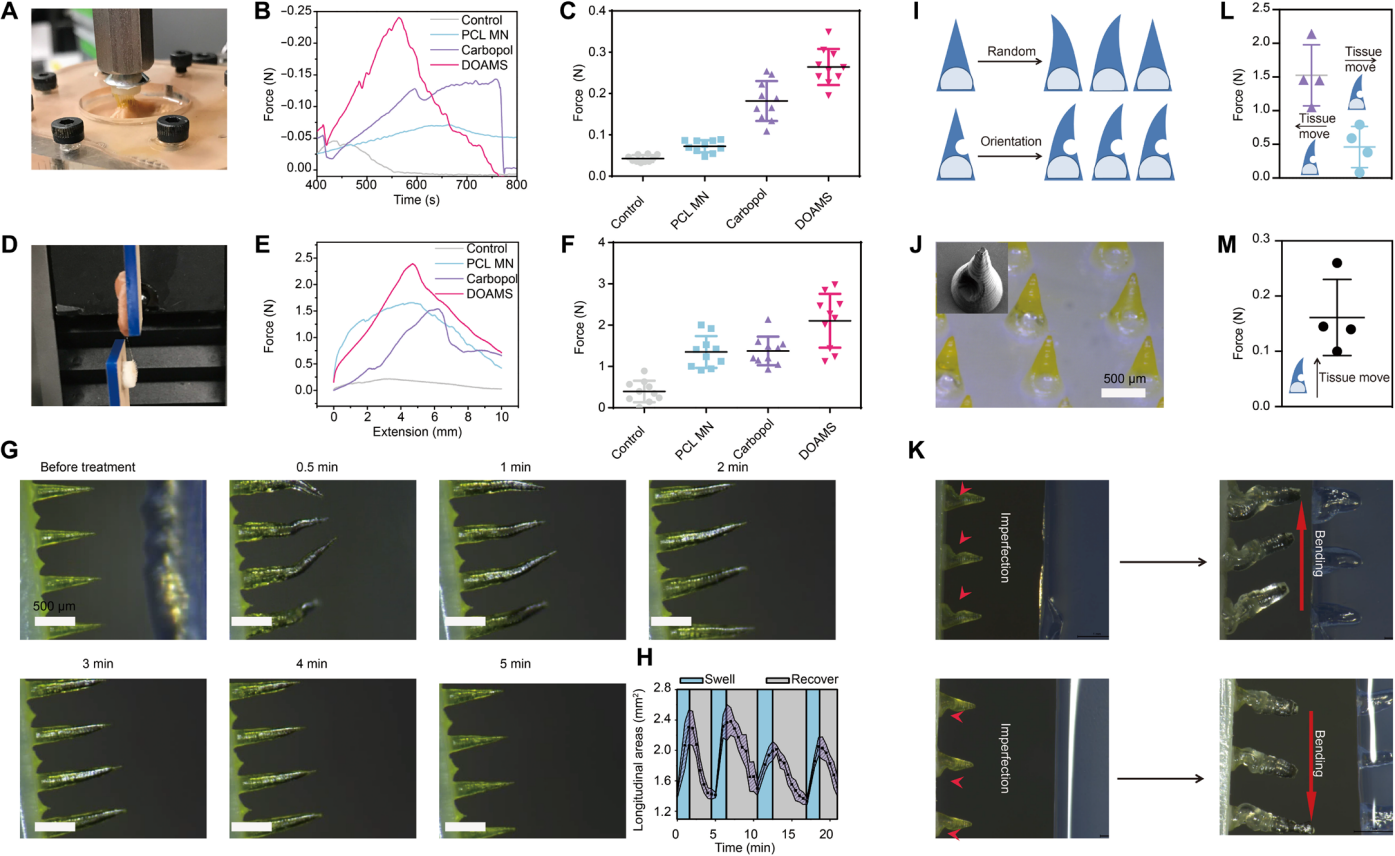
Adhesion test for dynamic microneedle systems on stomach tissue. (**A** to **C**) Pull-out analysis for different systems on pig stomach after 1 N of compression force for 5 min. Photo credit: Wei Chen, MIT. (**D** to **F**) The lap-shear test for different systems on the stomach tissue. Photo credit: Wei Chen, MIT. (**G**) Video screenshot displaying the recovery process of the DOAMS after swelling. Photo credit: Wei Chen, MIT. (**H**) Four swelling and recovery cycles of the DOAMS. (**I**) Schematic of orientation control for a DOAMS microneedle and an SDSM. (**J**) Microscope and SEM images of the imperfection on the SDSM. Photo credit: Wei Chen, MIT. (**K**) Bending control of the SDSM via the imperfection. Photo credit: Wei Chen, MIT. (**L**) Lap-shear test of the SDSM. (**M**) Pull-out test of the SDSM.

To quantitatively evaluate whether the DOAMS could adequately adhere to a target tissue, we performed pull-out and lap-shear tests on a swine stomach ex vivo using a uniaxial material testing machine (Instron 5943) (*32*). Using a circular punch, the DOAMS was cut into a round shape (diameter, 5 cm; 30 needles) and connected to the fixture of the machine. It was pressed into the stomach tissue for 5 min with a force of 1 N and then lifted at a constant speed of 1 mm/min. A cellulose pill (0.04 N) and a PCL MN (0.08 N) did not noticeably adhere to the tissue, potentially due to the weakness of the hydrogel bonds and interlock effects in the vertical direction. However, the DOAMS exhibited a remarkably high adhesive force toward the tissue (0.25 N), which was even stronger than commercial Carbopol powders (0.18 N) (Fig. 3, A to C). This is likely because there is more than one interlocking mechanism between the DOAMS and the tissue: the mechanically driven hook and the chemically based adhesion of the hydrogel bond between the Carbopol and the tissue. In a lap-shear test, the DOAMS resisted motion-induced shear stress better than the other systems (the shear force was around 2 N), although the PCL MN displayed considerable strength likely due to its orientation (the shear force was around 1.65 N) (Fig. 3, D to F). In addition, real-time observation revealed that the swelling process of the DOAMS occurred within 30 s but was able to recover gradually and spontaneously within 5 min (movie S2). Eventually, the DOAMS was restored to its original state, supporting a dynamic behavior profile with reversibility. Notably, the DOAMS could undergo at least four swelling and recovery cycles without losing significant adhesive strength; this suggests that it was flexible enough to remain fixated in dynamic tissue (Fig. 3, G and H). Note that this random and multidirectional bending is essential for strong tissue adhesion in both the normal and shear directions. Next, the interlocking behavior of the DOAMS microneedles was compared to that of a single-directional swelling microneedle (SDSM). To create an SDSM, we altered a DOAMS microneedle with a hemisphere-shaped imperfection (diameter was around 400 μm) on one side of the microneedles, which altered the orientation of the extension (Fig. 3I). As indicated in Fig. 3J, the imperfection was precisely constructed on the boundary of the PCL and Carbopol layers such that the orientation of the extension would be continuously altered during the entire inserting process. After penetrating the hydrogel, the SDSM swelled toward the imperfection no matter which direction it faced (Fig. 3K) since the pressing force was not uniformly distributed because of the asymmetric architecture. The SDSM exhibited a high shear strength when the force was applied in the opposite direction of bending as opposed to the same direction of bending (Fig. 3L). However, the normal adhesive strength of the SDSM was similar to that of the DOAMS microneedle (pull-out test; Fig. 3M). Since tissue movement is often dynamic and can occur in multiple directions and the shear adhesive strength of SDSM is inadequate for certain directions, multidirectional DOAMS would provide broader stability across a range of orientations of shear stress. The development and characterization of SDSM also support our capacity to control the direction of deformation in microneedle substrates.

### Jack-in-the-box device design

**Fig. 4.**
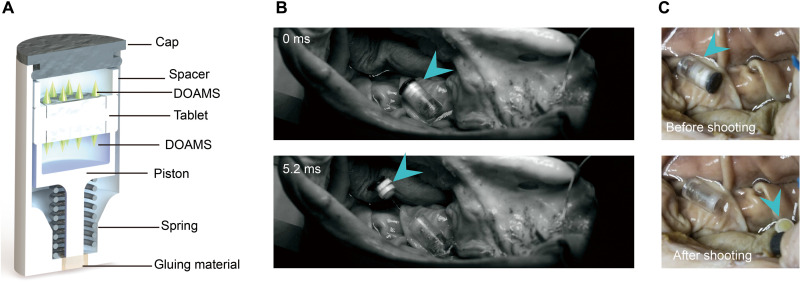
Jack-in-the-box–inspired capsule device prototype design and ex vivo test. (**A**) Design of the device containing the double DOAMS-modified tablet. (**B**) Images from a high-speed camera showing the successful trigger of the device. Photo credit: Jacob Wainer, MIT. (**C**) Images of the status of the double DOAMS-modified tablet before and after actuation. Photo credit: Jacob Wainer, MIT. Data clearly showed that one side of the DOAMS formed an appropriate contact with the tissue after being deployed.

To avoid premature exposure of a drug/formulation to the harsh GI environment, it is typically encapsulated in a gelatin capsule, which dissolves in the presence of GI fluid to release the payload. However, the dissolved gelatin can become extremely sticky, which can foul the dosage form and delay the release of the drug/formulation and prevent it from immediately contacting the tissues (*30*, *31*). Preexposure of the DOAMS would trigger early shape alteration, which would significantly reduce its mechanical strength (fig. S3), compromising effective tissue penetration and adhesion. To prevent preexposure, we developed a tube-shaped, self-triggered jack-in-the-box–inspired device to house the DOAMS-modified tablets (a spacer was used to protect the DOAMS microneedles; Fig. 4A). This jack-in-the-box device responds to acidic GI fluid to eject the tablet directly toward the tissue surface thereby minimizing GI fluid preexposure. Inspired by the traditional jack-in-the-box toy, a spring is held compressed by specific gluing materials (e.g., sugar glass) that dissolve in a humid and moist environment; once actuated, the spring pushes the tablet out of the device at a high speed (1.6 mm/ms) (Fig. 4B and fig. S4). In addition, to ensure that the microneedles make contact with the tissue (single DOAMS-modified tablet may induce unsuitable contact with microneedles of the tissue; fig. S5), the tablet was modified by two DOAMSs, one on each side. In addition, a spacer was placed between the piston and the DOAMS to provide enough space to protect the microneedles (Fig. 4A). Prior work has identified the stomach as the main site for SNAC-mediated semaglutide delivery (*18*). Our device was designed to be triggered by the humid gastric environment to deploy the engineered tablet onto the tissue surface (movie S3) while avoiding preexposure to the potentially fouling environment (Fig. 4C). Moreover, the gastric cavity also offers the potential for reduced variability in delivery, as the time to reach the stomach is much less variable than gastric exit (*33*). Note that once in contact with the tissue surface, not all DOAMSs could insert into the tissues, as the tablet may show an improper contact (angled or vertical form), which might retard the DOAMS-based mucoadhesion. We developed a simplified manually operated fixture (fig. S6, A and B) to evaluate and support optimization of tablet configuration. Optimal tissue interaction was determined by the degree of tissue interaction with MNs (larger contact areas indicated higher scores 1, 2, and 3; fig. S6C). Traditional tablets (with cavity but no DOAMS) were able to form satisfactory contact with the tissue (65 to 85%, score 3) when administered at both 0° and 45° angles. Tablets with taller needles (3.0 or 1.8 mm from tip to tablet surface; fig. S7) did not perform as well on the contact tests for any angles (10 to 45%, score 3). In comparison, tablets with needles that were 1.3 mm tall performed the best out of all the DOAMS-modified tablets: More than 50% of these tablets formed an ideal contact for both angles tested (fig. S6, D to G). In this regard, the shorter needle projections were better suited for the DOAMS design because they enabled the tablet to form satisfactory contact with the tissue more consistently.

### In vivo oral drug delivery

**Fig. 5.**
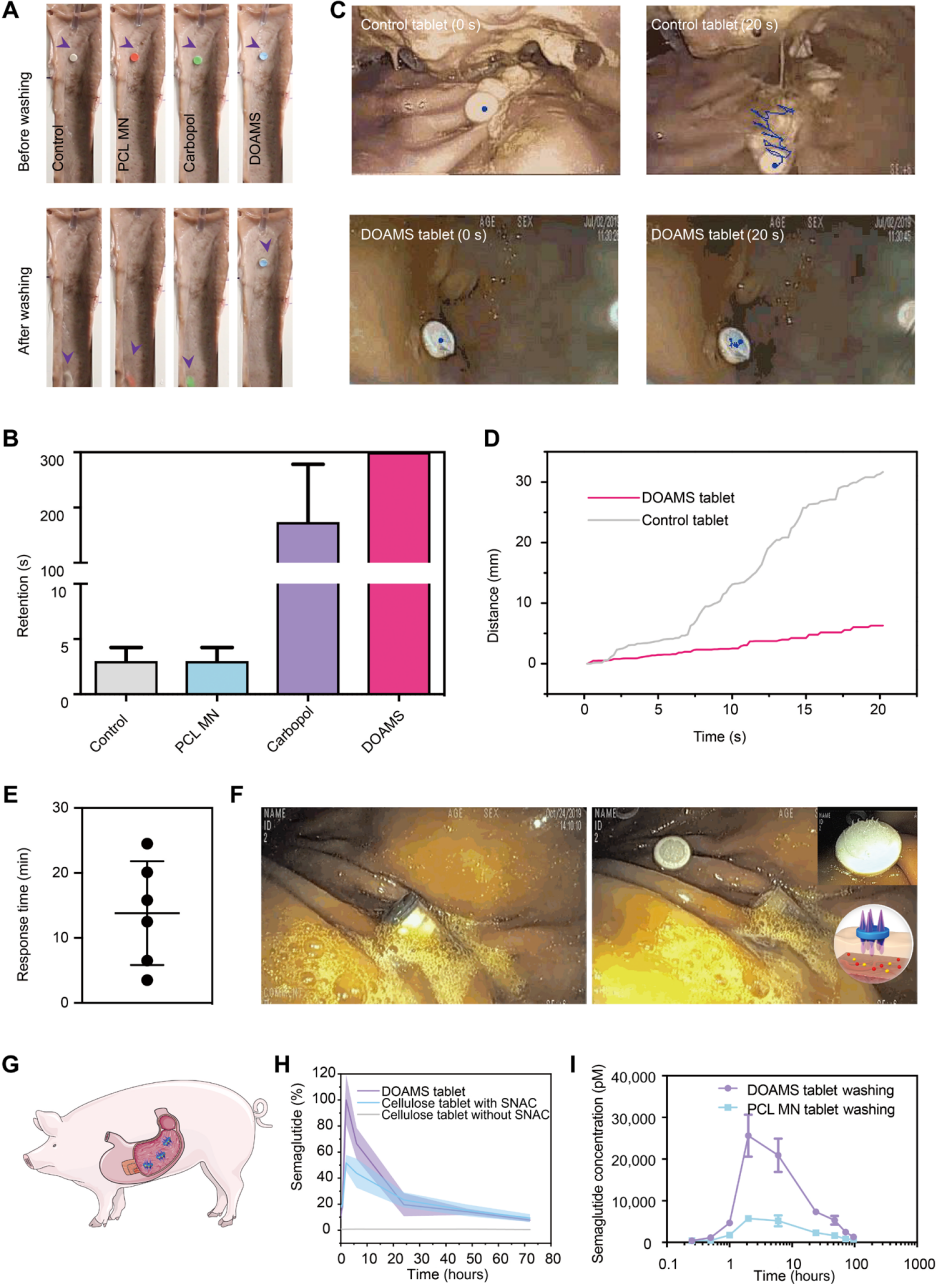
In vivo adhesion test. (**A**) Washing test on the tissues. Photo credit: Wei Chen, MIT. (**B**) The retention time of different tablets on the pig stomach tissues. (**C**) Endoscopic images showing the movement of the control and the single DOAMS-modified tablet in the pig stomach. Photo credit: Siddartha Tamang, MIT. (**D**) Distance traveled by the control and the single DOAMS-modified tablet in the pig stomach. (**E**) In vivo observation of actuation time for the jack-in-the box device. (**F**) Endoscopic images showing preactuation (left) and postactuation (right) of the jack-in-the-box device in the pig stomach. Photo credit: Siddartha Tamang, MIT. (**G**) Schematic for tablet delivery to pig stomach. (**H**) The arithmetic mean plasma concentration–time profiles of semaglutide after a single dose of oral semaglutide (10 mg) with 300 mg of SNAC in healthy pigs. (*n* = 3 to 5). Cellulose tablet without SNAC was set as a control. (**I**) The arithmetic mean plasma concentration–time profiles of semaglutide after a single dose of oral semaglutide (10 mg) with 300 mg of SNAC in healthy pigs and washed by using 15 ml of water.

To determine the mucoadhesion performance of the DOAMS-modified tablets compared to a set of controls, we applied an ex vivo tissue apparatus with controlled water wash (fig. S8). Specifically, DOAMS mucoadhesion was compared to a control tablet (Cellulose), a nonmucoadhesive microneedle-containing tablet (MN PCL), and a Carbopol tablet (with no microneedles). As shown in Fig. 5 (A and B), the control and the PCL MN–modified and the Carbopol-modified tablets were dislodged within 5 min under running water at 99 ml/min. The DOAMS-modified tablets demonstrated prolonged retention (in vivo washing test; fig. S9). To further characterize the mucoadhesive performance of DOAMS in vivo, we delivered DOAMS-modified tablets in the swine stomach and exposed these to a water wash test with concomitant endoscopic tracking and compared to controls (Fig. 5, C and D, and fig. S9). Using real-time endoscopic imaging, it was observed that traditional tablets (nonmucoadhesive and nonmicroneedle) migrated more than 30 mm within 20-s tracking, while DOAMS-modified tablets appeared to remain firmly in place (Fig. 5, C and D). To evaluate the deployment of a DOAMS-modified tablet from the jack-in-the-box prototype vehicle, we delivered these through endoscopic assistance to the stomach in vivo and monitored under endoscopic imaging (movie S4). Autonomous deployment secondary to the actuation of the vehicle by the humid environment was quantified through endoscopic visualization and observed to range from 4 to 25 min (Fig. 5E). The deployed DOAMS-modified tablets were observed to engage the gastric tissue with an appropriate contact angle (Fig. 5F), followed by secure adhesion. Delivery of semaglutide was compared between DOAMS-modified tablets and cellulose tablets. Both tablets were formulated with 10 mg of semaglutide (pure drug; fig. S10) and 300 mg of SNAC and delivered to the pig stomach (Fig. 5G). Semaglutide plasma concentration was measured using an AlphaLISA assay. Notably, compared to traditional cellulose tablets, the DOAMS-modified tablets exhibited a significantly higher absorption in pigs (Fig. 5H). This was likely supported by the stronger tablet-tissue interaction achieved by the DOAMS, which allowed the drug to concentrate on the interface between the tablet and tissues, facilitating the delivery of the enhancer and drug. The C_max_ (peak plasma concentration) after DOAMS-modified tablet treatment was around two times that of a traditional cellulose tablet treatment (Fig. 5H and table S1); T_max_ was comparable between the two. Consistent with prior work, SNAC was essential for semaglutide absorption, as the SNAC-absent group displayed low drug levels (Fig. 5H and table S1). In addition, since tablet ingestion is commonly paired with water intake, the effect of water flow on drug absorption was evaluated over the course of 3 days. As expected, ingestion of the adhesive tablets (DOAMS tablets) resulted in a much higher drug concentration in the blood (six times) compared with nonadhesive tablets (Fig. 5I). This preclinical data demonstrated that the DOAMS modification could be used to enhance the delivery of therapeutics by extending the drug-tissue interaction with minimal side effects.

## DISCUSSION

Here, we describe a delivery system that combines physical with nonphysical modes of delivery to enable the enhanced absorption of semaglutide. Moreover, we describe a tablet-deploying device capable of deploying tablets in the gastric cavity to minimize fouling predeployment. We demonstrate how omnidirectional bending microneedles have the potential to provide improved tissue interaction and, through coupling to mucoadhesives as penetration enhancers, can support enhanced delivery, which is transiently resistant to water follow. Given the challenges associated with food effects with respect to physically disturbing the development of transient privileged regions for enhanced transport systems (*18*), the capability of withstanding the physical interaction with ingested matter could support a reduction in variability of absorption of drugs. We and our collaborators have previously developed a series of systems to enhance oral drug delivery, ranging from contraceptives to biologics (insulin) (*15*, *16*, *34*). Here, we describe a system capable of supporting the delivery of a biologic through the enhancement of a microneedle patch that can interface and transiently fix itself through physical interaction to the mucosa to support enhanced delivery of a biologic.

Although the DOAMS and the jack-in-the-box device demonstrated promise in this initial large animal demonstration, there are several areas that will be critical to address toward supporting effective and successful clinical translation. Notably, the materials used to fabricate the tube-shaped capsules were mainly resins, which are not rapidly degradable. Biodegradable materials could provide alternatives with optimal safety and environmental impact profiles. The DOAMS has been shown to be resistant to water washing, but its tolerance across diets will be important to evaluate to guide dosing parameters. Last, only a single dose was administered in our test to evaluate the drug pharmacokinetics. The long-term effects of our system with respect to mucosal interaction and efficacy for the treatment of diabetes in preclinical models or in human subjects will be important. Successful translation of the DOAMS system will involve further validation of safety and efficacy studies conducted in large animal models including dog, pig, and, potentially, nonhuman primates, leading to first-in-human safety studies. From a safety perspective regarding GI elimination, the subcomponents of the device have been maintained below the recognized dimensions of the osmotic-controlled release oral delivery system (OROS), which has a complication rate of <1 in 76 million distributed tablets and is part of multiple successful Food and Drug Administration–approved drugs (*35*). From a scalability perspective, the devices will require manufacturing, which involves injection molding expertise and optimal quality control of solid dosage form analysis.

In summary, here, we report the combination of a range of recognized modes of drug delivery and demonstrate how these could be applied to the challenge of oral delivery of macromolecules. We anticipate that this system and its components could be applied across a range of applications including systems that require transient GI fixation for sampling and stimulation and delivery in specific GI compartments while mitigating transient exposure and fouling.

## MATERIALS AND METHODS

### Study design

The objective of this study was to develop a smart oral drug delivery system to prevent early drug exposure in the GI tract and improve mucoadhesion of the formulation to enhance delivery efficiency. First, the DOAMS was fabricated with a softer outer layer and a rigid inner layer to provide it with both penetration and extension capabilities. Swine stomach tissue was used to evaluate the mucoadhesive potential of the DOAMS, as well as its tolerance against water washing. To prevent early exposure, a jack-in-the-box device was invented to house the DOAMS-modified tablets during oral administration. During in vitro testing, a high-speed camera was used to record the actuation details of the device, and an optimization study was conducted to determine the height of the microneedles that would lead to optimal contact (higher contact area) of the DOAMS-modified tablets on the swine stomachs. Real-time endoscopy was used to investigate the in vivo behavior of the devices; the semaglutide and a penetration enhancer, SNAC, were codelivered to evaluate the ability of the DOAMS and the jack-in-the-box device to improve delivery.

### Fabrication of the DOAMS

Three-dimensional (3D) DOAMS molds (master) with or without imperfection were designed in SolidWorks (Dassault Systèmes, Vèlizy-Villacoublay, France) and then printed with gray resin on a Form2 3D printer (Formlabs, Somerville, USA) or MicroFine green resin (Protolabs, Maple Plain, USA). A SYLGARD 184 (Dow Inc., Midland, USA) PDMS mold (negative) was fabricated from the master mold by mixing two-part resin systems containing vinyl groups (part A) and hydrosiloxane groups (part B), and the material was cast around the master. After cross-linked network formation, the flexible elastomeric molds were carefully removed. Carbopol 971P NF (Lubrizol, Wickliffe, USA) polymer was dissolved in dimethylformamide under sonication (100 kHz) with a concentration of 25 mg/ml. FITC (5%) was added to the Carbopol solution as an indicator. After weighing and balancing the molds, 200 μl of Carbopol solution was added, followed by 10 min of vacuum (600 mmHg) treatment. After centrifugation (2000 rpm for 10 min; Eppendorf AG 22331 Hamburg, Germany), the molds were placed in a desiccator for another 10-min treatment and then placed in a vacuum chamber for 24 hours at 45°C. The casting process was repeated between one and five times. Subsequently, 50 mg of PCL (45 kDa; CAPA 6400, Perstorp) was introduced to the mold, followed by heating to 120°C; the mold was then placed in a vacuum for 3 hours. After cooling to room temperature, the MN arrays were carefully peeled off from the PDMS mold and maintained at room temperature for further applications.

### Fabrication of the tablets

The tablets were compressed using an NP-RD10A tablet pressing machine (Natoli Engineering, Saint Charles). Each tablet contained around 10 mg of semaglutide, 300 mg of SNAC, and 400 mg of polymer matrix (carboxymethyl cellulose). The powder blender was mixed using a FlackTek SpeedMixer (Landrum, SC) at 1500 rpm for 30 s. During the pressing, the force was 12,000 N, and the diameter was set to 8 mm. All tablets were stored at −20°C until use. Tablets that contained polymer alone were set as a control group.

### Jack-in-the-box capsule device

The device was designed in SolidWorks (Dassault Systèmes, Vèlizy-Villacoublay, France). The capsule and piston components were 3D printed using VeroClear resin with an Objet30 Pro (Stratasys Ltd., Rehovot, Israel). Spacers, which prevented the DOAMS microneedles from being damaged when stored, were lasercut using a VLS6.60 CO_2_ laser cutter (Universal Laser Systems, Scottsdale, USA) and Illustrator (Adobe Inc., San Jose, USA). The spacers were cut from PTFE (polytetrafluoroethylene) sheeting (McMaster-Carr, Elmhurst, USA) with a thickness based on the height of the needles. The device’s cap was 3D printed with flexible resin on a Form2 (Formlabs, Somerville, USA) 3D printer. Stainless steel compression springs (Lee Spring, New York, USA) were custom-made with an outer diameter of 3.7 mm, a wire diameter of 0.018 in, the free length of 12.28 mm, and a rate of 1.307 N/mm. First, the piston was glued using medical-grade Loctite adhesive (Henkel, Düsseldorf, Germany) to one end of the spring. Next, the free end of the spring was glued to the bottom of the inside of the capsule. Using a heat gun, a bead of isomalt (Sigma-Aldrich, St. Louis, USA) was melted on the polished surface of a mirror plate. Once liquefied, the capsule was placed over the isomalt, and the stem of the piston was pressed through the spring and capsule into the molten isomalt. The device was held in place until the isomalt solidified, locking the spring in a compressed state. The DOAMS-modified tablet was placed in the capsule, and a spacer was placed around the upward-facing DOAMS. Once the internal components were flush, the cap was placed on top of the capsule.

### Agarose hydrogel penetration

Aiming to visualize the shape alteration of the DOAMS during the treatment, a 0.5 to 1.5% agarose hydrogel was prepared to mimic a wet and soft stomach mucus layer. After cutting between microneedle rows, a single array of MNs was linked to a fixture, which could help adjust the height flexibly. Meanwhile, the agarose hydrogel was tailored to a flat shape for facilitating the focus of the microneedle. The swelling and recovery processes were observed using a Leica M165 C stereo microscope with a coupled Leica DFC450 camera (Leica Microsystems, Buffalo Grove, IL, USA).

### Tissue adhesion test

In the pull-out test, harvested tissue was affixed to a plastic fixture with a 2.5-cm-diameter hole in the center. The fixture was lasercut to size on a VLS6.60 CO2 laser cutter (Universal Laser Systems, Scottsdale, USA) out of 0.625-cm-thick acrylic sheeting (McMaster-Carr, Elmhurst, USA) and assembled with hardware purchased from McMaster-Carr. The control tablet, PCL MN, Carbopol, SDSM, or DOAMS was connected to the Instron 5943 machine’s cross-head. After maintaining a force of 1 N in compression for 5 min, the cross-head was lifted at a rate of 0.1 mm/s until a force drop event occurred. In lap-shear measurement, the tissue and tablet/MN were glued (Loctite) to opposite rectangular acrylic sheets (McMaster-Carr) that were lasercut to size on a VLS6.60 CO2 laser cutter (Universal Laser Systems) and stabilized vertically using screw side-action grips (Instron). After adjusting the distance to align tablet/MN contact with the stomach, the tissue was raised at a speed of 0.1 mm/s. All perforation events were correlated to a force drop.

### High-speed video recording

When the DOAMS-modified tablet was loaded into the jack-in-the-box device, it was placed horizontally or directly on the stomach tissues. An Edgertronic SC2 high-speed camera (Sanstreak Corp., San Jose, USA) was used to observe actuation events at 10,000 frames/s. Tablets were observed until the desired ballistic motion had ceased or were out of frame.

### Optimization for the contact of DOAMS on tissues

The DOAMS was punched to a circular shape (5 mm in diameter) and attached to an 8-mm-diameter tablet using a medical device Loctite adhesive (Henkel, Düsseldorf, Germany). The base of the MN was sanded using a Grizzly H6070 belt sander (Grizzly Industrial, Bellingham, USA) to regulate the height between the needle tip and tablet surface. Material removal from the base was controlled with a 3D printed fixture printed on an Objet30 Pro (Stratasys Ltd., Rehovot, Israel). The tablets were milled using HSMWorks (Autodesk Inc., San Rafael, USA) and an Othermill benchtop CNC mill (Bantam Tools, Peekskill, USA) to construct a cylinder-shaped cavity to load the DOAMS. The tablets were affixed in the Othermill with a laser-cut VLS6.60 (Universal Laser Systems) piece of acrylic (McMaster-Carr). Candidates (3.0, 1.8, and 1.3 mm) were tested using a manually operated device with two angles (0° and 45°). The manual device was printed using an Objet30 Pro (Stratasys Ltd., Rehovot, Israel) 3D printer for both the “cannon”-like section and the angle block component. Vertical, angled, and horizontal contacts were scored as 1, 2, and 3, respectively. On the basis of 20 repeats, their contact was statically analyzed.

### Detachment test

A flow model apparatus was used to examine the retention effect of the fabricated device following our previous method (*32*). Excised porcine stomach tissues (Lemay and Sons, Goffstown, USA) were tailored to a length of 25 cm and a width of 8 cm and then placed onto a specific apparatus (fig. S8) with the mucosal side facing up. The tablets, which were labeled with different colors, were placed onto the tissues 4 cm from the top of the slides. After setting the tilt angle of 45°, the mucosal surface was continuously flushed with water at 99 ml/min. The times for dislodgment were recorded, and videos were archived with a Canon EOS 5DS DSLR camera (Canon Inc., Tokyo, Japan).

### In vivo evaluation of the DOAMS system

All animal experiments were approved by, and performed in accordance with, the Committee on Animal Care at Massachusetts Institute of Technology (MIT). To evaluate the effect of the jack-in-the-box on DOAMS-modified tablet delivery, Yorkshire swine (Tufts University, Grafton, USA) of 35 to 65 kg was administered orally by the designed prototype. Before the delivery, the swine was placed on a liquid diet for 24 hours and fasted overnight. Then, the animal was sedated by intramuscular injection of Telazol (tiletamine/zolazepam) (5 mg/kg), xylazine (2 mg/kg), and atropine (0.05 mg/kg), along with supplemental isoflurane (2% in oxygen at 2 liter/min) via a face mask. An overtube from the mouth, through the esophagus to the stomach, was used to facilitate the passage of the device. A gastric endoscope was applied to visualize the device and tablets. A video was used to document the actuation process, as well as the sequential photographs. The movement of the tablets was analyzed on the basis of the videos by using ImageJ software.

We collected blood samples via a central venous line into ethylenediaminetetraacetic K3 tubes (Sarstedt, Newton, USA) at various time points, including (but not limited to) 0, 0.25, 0.5, 1, 2, 6, 24, 48, and 72 hours. Immediately, the blood glucose level was measured using a OneTouch Ultra glucose monitor by LifeScan. Subsequently, the blood was centrifuged (4000 rpm for 10 min), and the plasma was analyzed via an AlphaLISA method developed by Novo Nordisk. Briefly, it included two latex bead reagents and a biotinylated antibody, which was one of the antibodies in the sandwich. During the assay, the three reactants combined with the analyte to form a bead-aggregate-immune complex. Illumination of the complex released singlet oxygen from the donor beads, which channeled into the acceptor beads and triggered chemiluminescence, which was measured in the Tecan plate reader.

## Notes

View/request a protocol for this paper from *Bio-protocol*.
