# Supplementary material for: Dynamic omnidirectional adhesive microneedle system for oral macromolecular drug delivery
Source: Sci Adv. 2022 Jan 5;8(1):eabk1792. doi: 10.1126/sciadv.abk1792 (PMC8730401; doi:10.1126/sciadv.abk1792)
Supplement: Supplementary file 1 — Figs. S1 to S10 Table S1 Legends for movies S1 to S4 [file sciadv.abk1792_sm.pdf]

Science Advances
AAAS

Supplementary Materials for

Dynamic omnidirectional adhesive microneedle system for oral macromolecular drug delivery

Wei Chen, Jacob Wainer, Si Won Ryoo, Xiaoyue Qi, Rong Chang, Jason Li, Seung Ho Lee, Seokkee Min, Adam Wentworth, Joy E. Collins, Siddartha Tamang, Keiko Ishida, Alison Hayward, Robert Langer, Giovanni Traverso*

*Corresponding author. Email: cgt20@mit.edu, ctraverso@bwh.harvard.edu

Published 5 January 2022, *Sci. Adv.* **8**, eabk1792 (2022)
DOI: 10.1126/sciadv.abk1792

The PDF file includes:

Figs. S1 to S10
Table S1
Legends for movies S1 to S4

Other Supplementary Material for this manuscript includes the following:

Movies S1 to S4

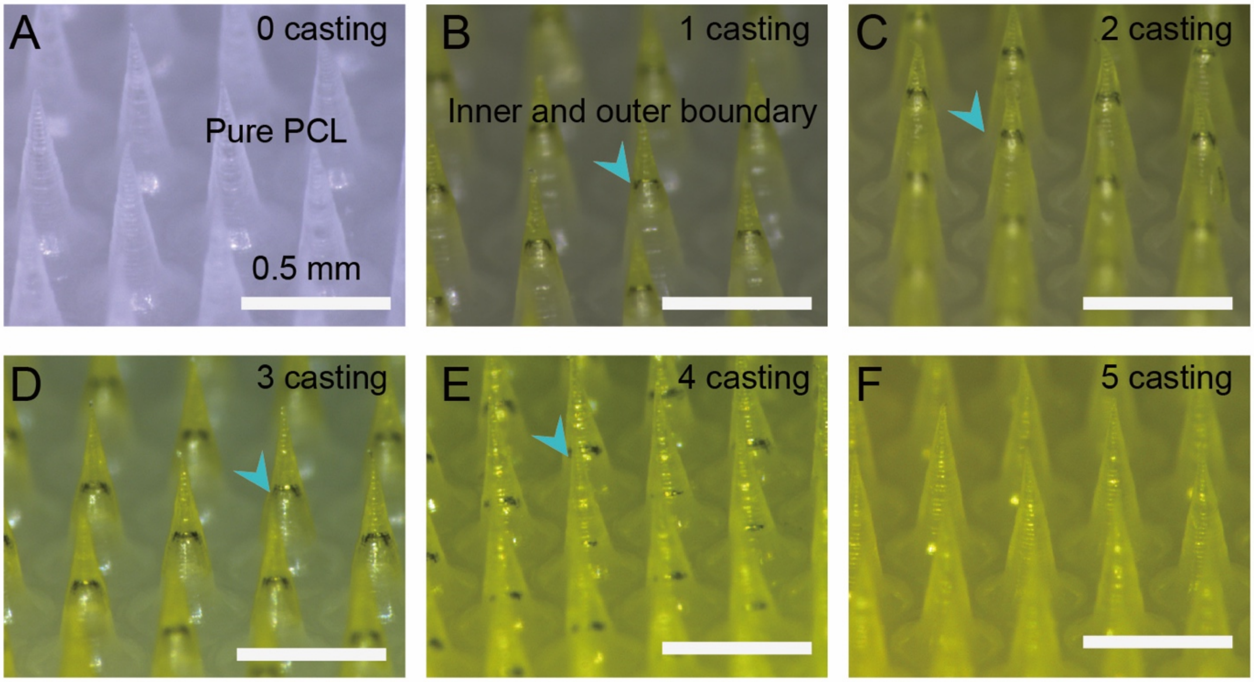

Fig. S1. **Microscope images of DOAMS with different Carbopol castings**. DOAMS fabrication consists of addition of the Carbopol solution to the PDMS mold for casting, followed by PCL addition. Pure PCL is white in color (A) while the Carbopol layer appeared yellow due to FITC inclusion (B-F). Blue arrows indicate the boundary between the outer and inner layers. As the number of Carbopol casting times increased, more Carbopol occupied the space in the needle (5 castings showed no PCL), suggesting that the ratio of PCL and Carbopol could be well regulated. It should be mentioned that dip coating on PCL microneedles would not construct this kind of core-shell structure as Carbopol could only form a quite thin layer on the PCL surface, and the shape was not easy to maintain when the system was not restricted in PDMS mold during dip coating. Therefore, the approach based on multiple castings in PDMS was the optimal method to fabricate the DOAMS microneedles. Photo credit: Wei Chen, MIT.

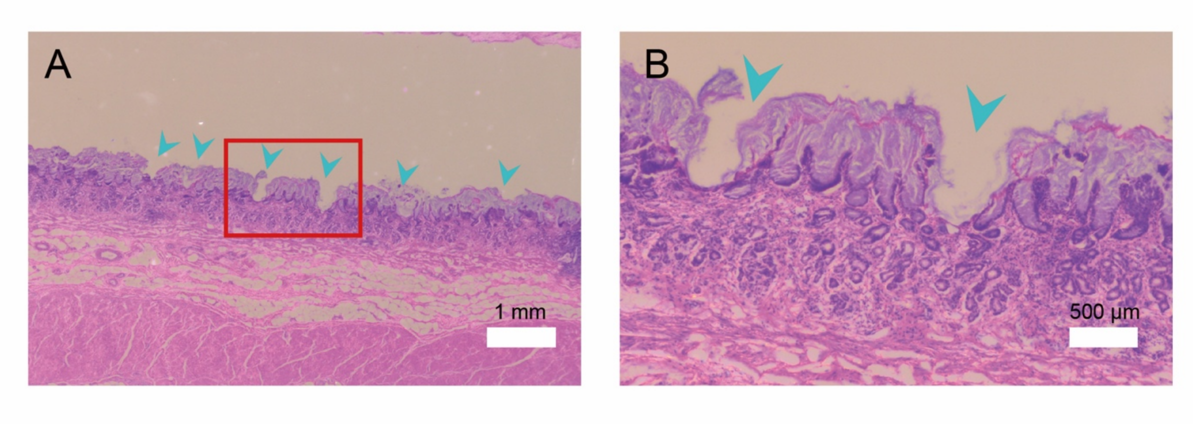

Fig. S2. H&E-stained section of pig stomach penetrated by DOAMS. Pig stomach was treated by DOAMS for 10 min, followed by fixation by paraformaldehyde. Then the tissues were paraffin-embedded, sectioned with a thickness of 15 µm, followed by H&E staining. (A) Microchannel arrays induced by DOAMS (blue arrows) were observed in the epithelium, indicating successful tissue penetration. (B) Magnification of the rectangle area in fig. S2A. Blue arrows indicate the microchannels. Photo credit: Wei Chen, MIT.

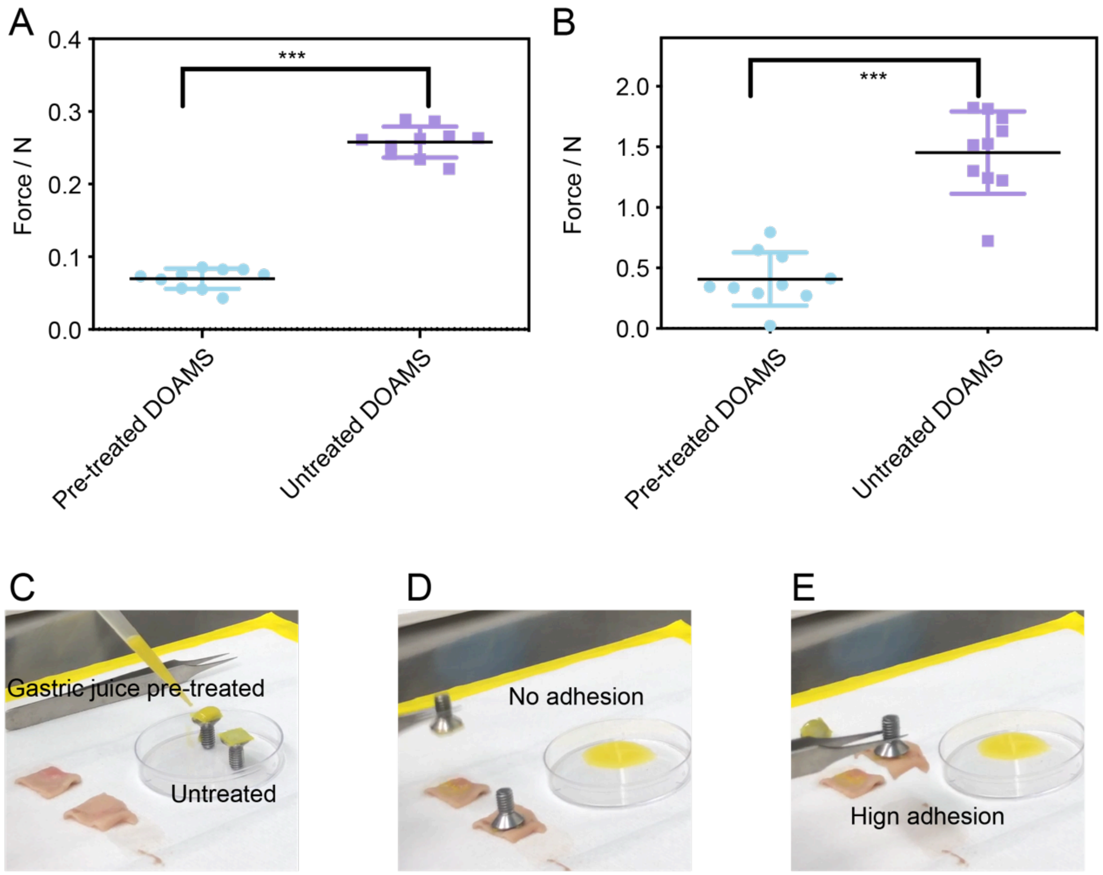

Fig. S3. **Pre-exposure to gastric fluid of DOAMS compromised DOAMS adhesion.** In order to assess the influence of gastric fluid on the mucoadhesive behavior of DOAMS, the DOAMS was pre-treated with porcine gastric fluid for 30 min, followed by pull-out and lap shear tests on a swine stomach *ex vivo* using a uniaxial material testing machine (Instron 5943). (A) The pull-out force of the DOAMS before and after gastric juice treatment. Mean ± SD (N = 10), ***$p < 0.001$. (B) The lap-shear strength of the DOAMS before and after gastric juice treatment. (C-E) Video screenshots displayed that pre-exposure to gastric juice reduced the adhesive capability of the DOAMS, which could not lift the stomach tissues. Photo credit: Wei Chen, MIT. These observations motivated the development of a system capable of deploying DOAMS intragastrically, see Fig. S4.

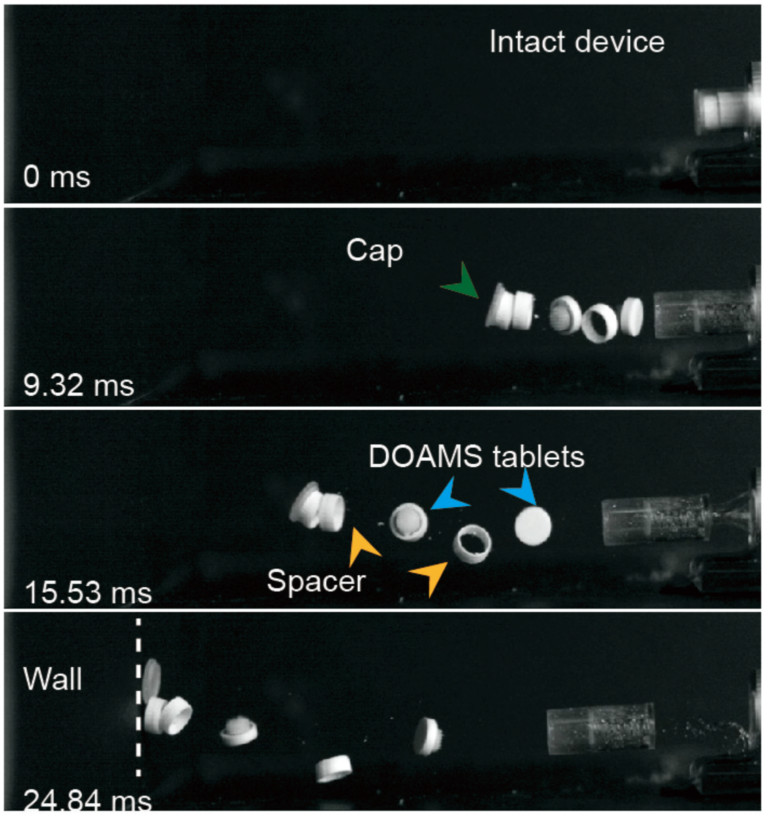

Fig. S4. Images from a high-speed video showing the actuation of the "Jack in the box" device. In one test, the intact device containing two single-DOAMS-modified tablets was placed horizontally on a fixture, followed by gastric fluid treatment at the end containing gluing materials. After approximately 20 min, the device spontaneously actuated and the spring pushed the tablet out of the device. An Edgertronic SC2 high-speed camera (Sanstreak Corp, San Jose USA) was used to observe actuation events at 10,000 frames/second. Within 9.32 ms, the cap (green arrow) and the spacers (yellow arrows) started to separate from the DOAMS-modified tablets (blue arrows). After 15.53 ms, they were completely separated. Photo credit: Jacob Wainer, MIT. The white dotted line marks a solid object to restrict further displacement.

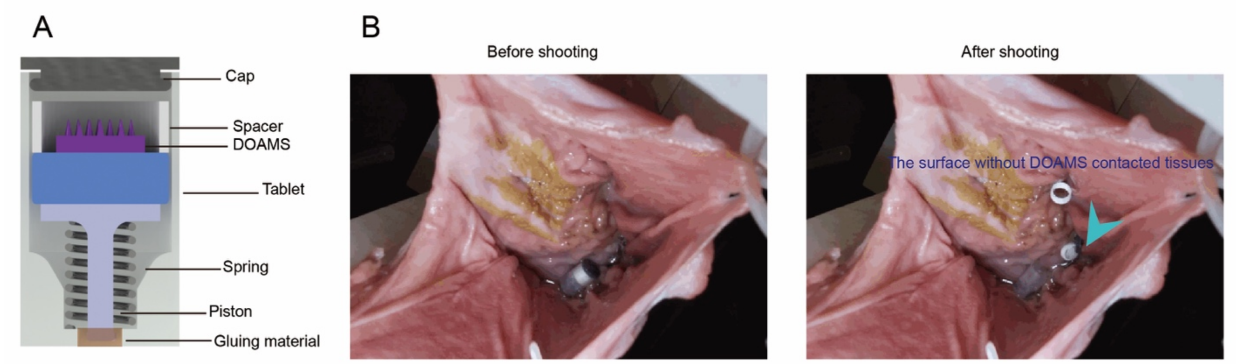

Fig. S5. Characterizing tissue contact of the single-DOAMS-modified tablet. After loading the single-DOAMS-modified tablet into the "Jack in the Box" device, this was placed in a pig stomach *ex vivo* to observe its actuation and the deployment of the tablet. After approximately 20 min, the device spontaneously actuated and the tablet was deployed out of the device. (A) Design of the "Jack in the Box" device containing a single-DOAMS-modified tablet. (B) Status of the device before and after deployment. The MN of the single-DOAMS-modified tablet faced up (blue arrow), which would not contact the tissue surface, indicating an unfavorable contact. Photo credit: Wei Chen, MIT. Therefore, the single-DOAMS-modified tablet could not guarantee that the surface of the tablet containing DOAMS could contact the tissues. This motivated formulation of double-sided DOAMS-modified tablets for subsequent studies.

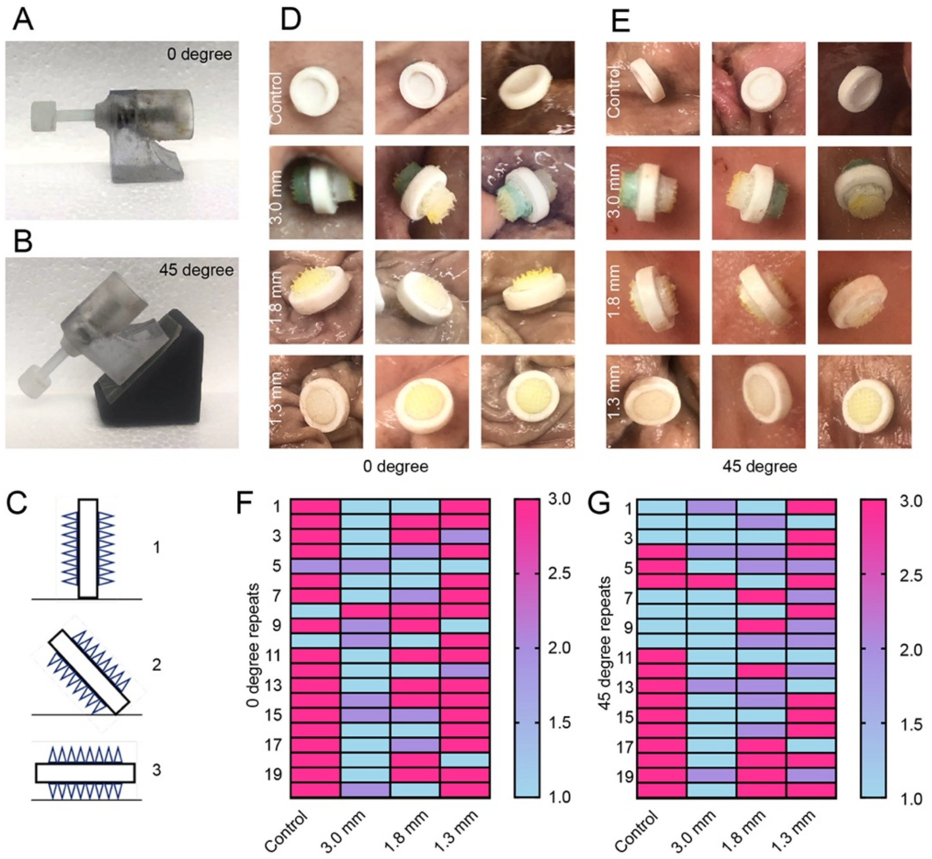

Fig. S6. Contact optimization for DOAMS-modified tablet. (A and B) Manually operated devices were used to test the ability of the tablets to establish good contact with the tissue as a function of the deployment angles (0 and 45 degrees). Photo credit: Wei Chen, MIT. (C) The scoring metric for the tablet contacting the tissue. (D and E) Images of different tablets after being deployed onto stomach tissue. Photo credit: Wei Chen, MIT. (F and G) Scores of different tablets based on the contact form with the tissue (N=20). Data showed that shorter MN indicated higher scores.

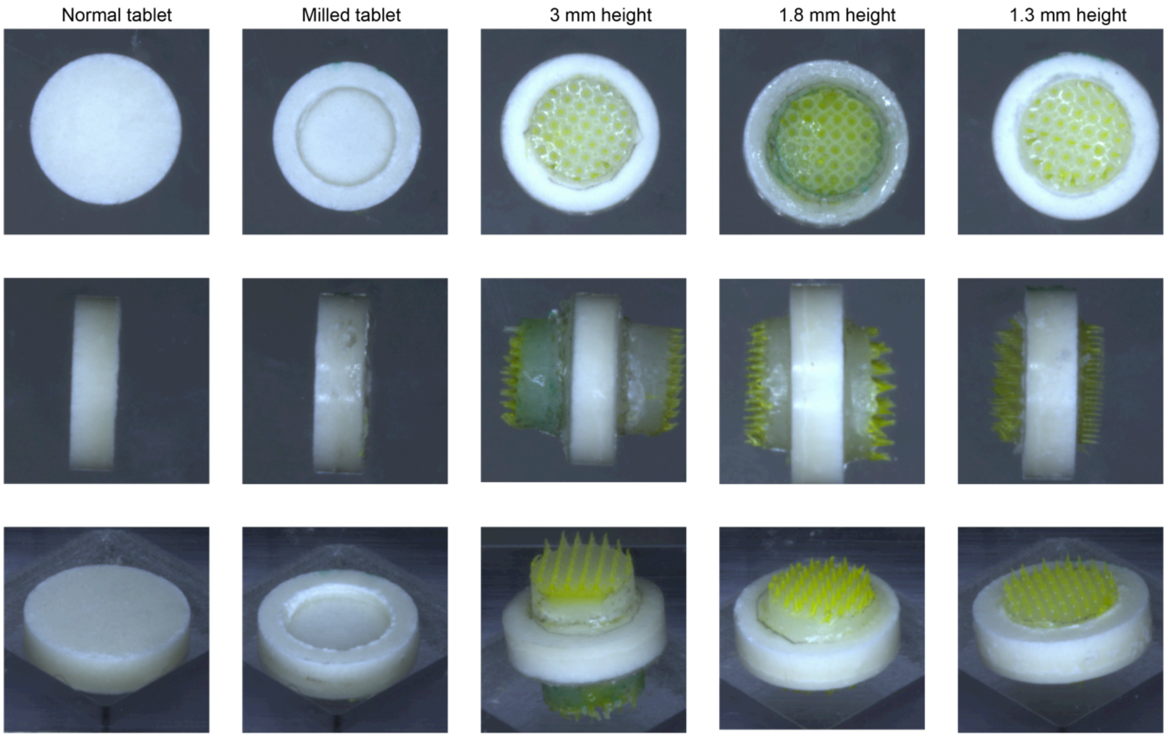

Fig. S7. **Tablet design and MN height optimization.** The height between needle tips and the tablet surface was explored through the milling of the tablet. The DOAMS was cut to a round shape with the same size (5 mm), which could be inserted into the cavity. The thickness of the base layer of the DOAMS could also be regulated by using a Grizzly H6070 belt sander, which could adjust the height of the DOAMS-modified tablet. Specifically, three kinds of height (3 mm, 1.8 mm, 1.3 mm) were tested in this study. Photo credit: Wei Chen, MIT.

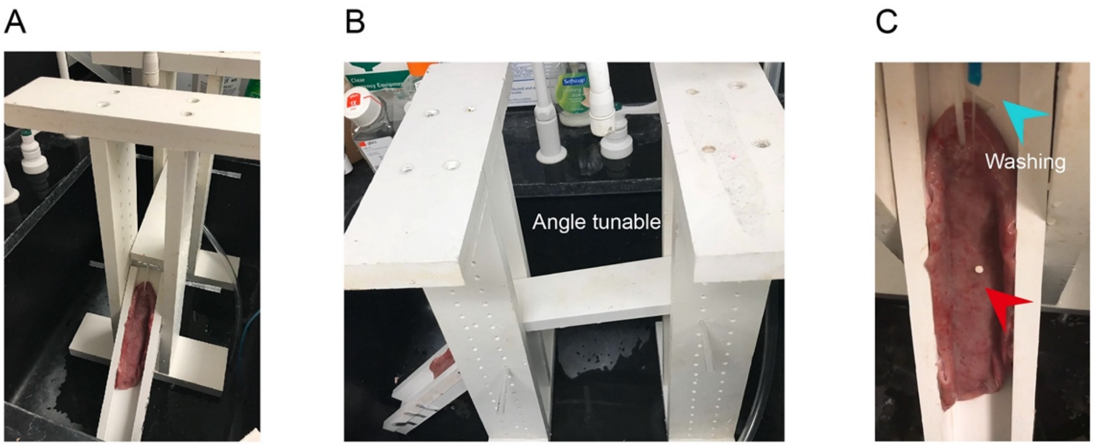

Fig. S8. Apparatus for *ex vivo* mucoadhesion washing evaluation. Excised porcine stomach tissues were tailored to a length of 25 cm and a width of 8 cm, and then placed onto the slope of the apparatus. After placing the tablet onto the tissue, the mucosal surface was continuously flushed with water at 99 ml/min. (A and B) Images of the apparatus from different angles. Photo credit: Wei Chen, MIT. (C) Picture showing the water stream (blue arrow), which could be controlled by using a pump. The red arrow indicates the tablet on the stomach tissue. Photo credit: Wei Chen, MIT.

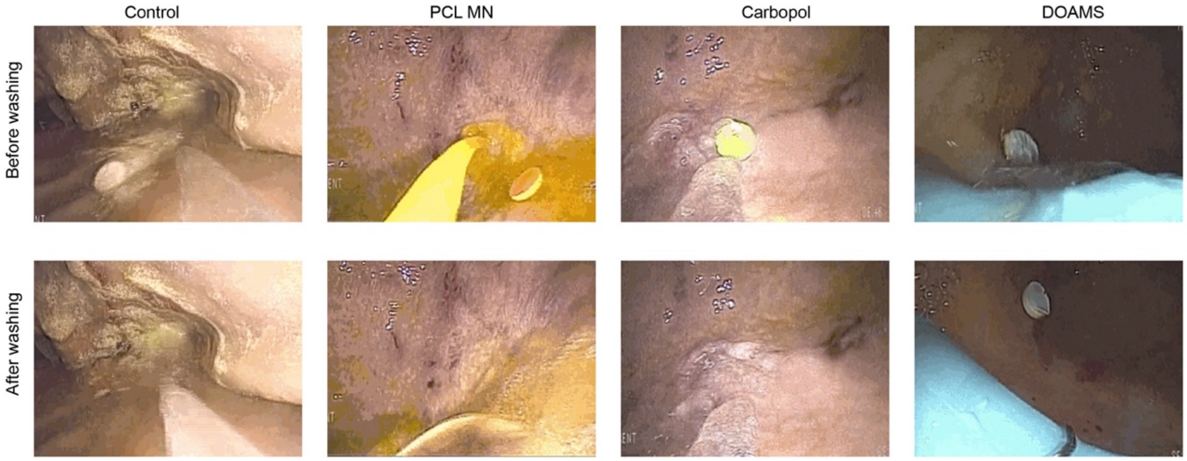

Fig. S9. ***In vivo* washing test of different tablets on pig stomach tissue.** A control tablet (Cellulose, white), a non-mucoadhesive microneedle-containing tablet (MN PCL, red), a Carbopol tablet (with no microneedles, yellow) and a DOAMS-modified tablet (blue) were delivered through endoscopic assistance to the stomach. A repeated water wash (15 ml) was applied to evaluate their retention capability. Photo credit: Joy Collins, MIT. Data significantly showed that the DOAMS-modified tablet (blue) resisted the water wash compared to blank pill (white), PCL MN-modified tablet (red) and Carbopol-modified tablet (yellow) after at least 5 rounds of washing treatment (they were gone after washing).

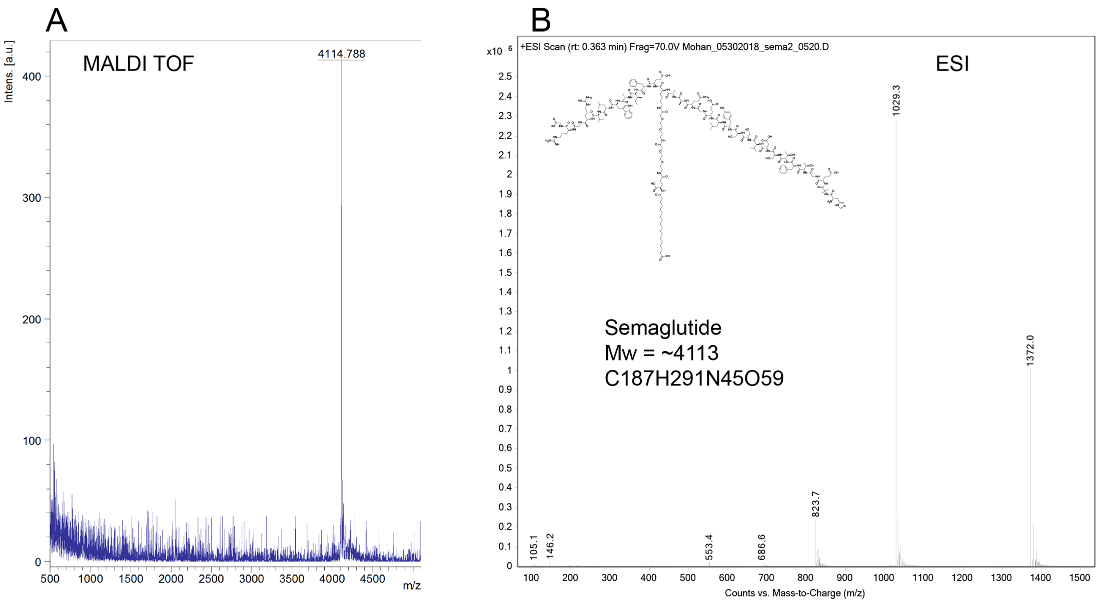

Fig. S10. **Mass analysis of semaglutide.** The molecular weight of semaglutide is around 4113, and MALDI TOF results displayed a single peak at 4114.788 (m/z), indicating a [semaglutide + H^+] signal. Moreover, the Electrospray ionization (ESI) scan showed a series of signals including [semaglutide + $3H^+$] (m/z, 1372.0), [semaglutide + $4H^+$] (m/z, 1029.3) and [semaglutide + $5H^+$] (m/z, 823.7). All these results confirmed the purity of the source material.

Table S1. Pharmacokinetics parameters in swine studies of semaglutide.

	Cmax (pM)	Tmax (h)	Area under the curve (pM·h)	t1/2 (elimination) (h)
**DOAMS tablet (N=5)**	33542.1±6023.2	5.2±0.9	592790.0±57583.4	26.7±4.1
**Cellulose tablet w/SNAC (N=3)**	17452.8±2045.4	5.0±0.6	510819.7±50099.6	25.4±3.9
**Cellulose tablet w/o SNAC (N=3)**	N/A	N/A	17064±2823.8	N/A

Movie S1. DOAMS spontaneously swelled in the hydrogel. When inserting into agarose hydrogel (1.5%), the DOAMS spontaneously swelled within 30 s.

Movie S2. Extension and recovery of DOAMS. Within 20 min, the DOAMS could swell in response to hydrogel treatment and recover spontaneously when separated from the gel.

Movie S3. Video from a high-speed camera indicated the deployment process of the device. When the device was placed in the pig's stomach, it could actuate spontaneously in response to the gastric fluid without any manual intervention. The cap, the spacer would separate from the DOAMS-modified tablet after shooting, thus these would not affect the tablet-tissue interaction.

Movie S4. *In vivo* actuation of the device. After delivering the device to the pig stomach *in vivo*, the system would actuate spontaneously in response to the gastric fluid to deploy the tablet onto the surface of the stomach. Then DOAMS helped the tablet transiently fix itself onto the tissue.
